# Clusterin is a potential molecular predictor for ovarian cancer patient's survival: targeting Clusterin improves response to paclitaxel

**DOI:** 10.1186/1756-9966-30-113

**Published:** 2011-12-20

**Authors:** Mohamed K Hassan, Hidemichi Watari, Yimin Han, Takashi Mitamura, Masayoshi Hosaka, Lei Wang, Shinya Tanaka, Noriaki Sakuragi

**Affiliations:** 1 Department of Obstetrics and Gynecology, Hokkaido University Graduate School of Medicine, Sapporo, 060-8638, Japan; 2 Biotechnology Program, Zoology Dept., Faculty of Science, Port Said University, Port Said, 24151, Egypt; 3 Laboratory of Cancer Research, Department of Pathology, Hokkaido University Graduate School of Medicine, Sapporo, 060-8638, Japan

**Keywords:** Clusterinn, Ovarian Cancern, Chemo-resistance

## Abstract

**Background:**

Clusterin is a cytoprotective chaperone protein involved in numerous physiological processes, carcinogenesis, tumor growth and tissue remodelling. The purpose of this study was to investigate whether clusterin (CLU), an antiapoptotic molecule, could be a potential predictor molecule for ovarian cancer and whether or not targeting this molecule can improve survival of ovarian cancer patients.

**Methods:**

Clusterin expression was compared between ten primary and their recurrent tumors from same patients immunohistochemically. We analyzed prognostic significance of CLU expression in another 47 ovarian cancer tissue samples by immunohistochemistry. We used small interference RNA to knock down CLU in the chemo-resistant ovarian cancer cell lines. KF-TX and SKOV-3-TX, paclitaxel-resistant ovarian cancer cells, were established from parental KF and SKOV-3 chemo-sensitive cell lines, respectively. Either siRNA or second generation antisense oligodeoxynucleotide against CLU (OGX-011), which is currently evaluated in clinical phase II trials in other cancer s, was used to modulate sensitivity to paclitaxel (TX) in ovarian cancer cells *in vitro*. Cellular viability assay, FACS analysis and annexin V staining were used to evaluate the comparative effect of CLU knocking down in ovarian cancer cells.

**Results:**

Immunohistochemical analysis of CLU expression in primary ovarian cancer tissue specimens and their recurrent counterparts from same patients demonstrated higher expression of CLU in the recurrent resistant tumors compared with their primary tumors. High expression of CLU by immunohistochemistry among 47 surgical tissue specimens of early-stage (stage I/II) ovarian cancer, who underwent complete cytoreduction as a primary surgery, significantly related to poor survival, while none of other clinicopathological factors analyzed were related to survival in this patient cohort. Secretory CLU (s-CLU; 60 KDa) expression was upregulated in TX-resistant ovarian cancer cells compared to parental cells. Transfection of siRNA or OGX-011 clearly reduced CLU expression. Cell viability assay, FACS analysis and annexin V staining demonstrated that targeting CLU expression by siRNA or OGX-011 sensitized ovarian cancer cells to TX.

**Conclusion:**

We conclude that CLU could be a potential molecular target to predict survival while targeting this s-CLU may improve survival of patients with ovarian cancer.

## Background

Chemotherapeutic drug resistance is a critical problem in cancer therapy as many tumors are intrinsically tolerant to some of the cytotoxic agents used, while others, although they are initially sensitive, recur and eventually acquire resistance to subsequent treatment with anti-neoplastic agents [1].

Ovarian cancer is the fourth common cause of cancer-related death in women because 75% of ovarian cancers are detected as late-stage disease [2,3]. Nevertheless, after optimal surgical debulking of the tumor and standard chemotherapy, patients with advanced disease experience 5-year survival rate [4]. Despite the relative sensitivity of ovarian cancer to chemotherapy, clinical chemotherapeutic treatment often encounters drug resistance [5]. Development of this acquired resistance represents the major limitation to successful treatment. Consequently, there is a pressing need to identify the mechanisms underlying resistance in order to develop novel drugs to re-sensitize tumor cells to primary chemotherapy.

Recently, histologic subtype has been recognized as one of the key factors related to chemosensitivity in ovarian cancer. Especially, clear cell carcinoma of the ovary, which is recognized as a distinct histologic entity in the World Health Organization classification of ovarian tumors, demonstrates a distinctly different clinical behavior from other epithelial ovarian cancers. Several studies showed that patients with clear cell carcinoma had a poor prognosis, partly due to a low response rate to chemotherapy [3-5]. However, little is known about the mechanisms of chemoresistance (intrinsic resistance) of clear cell carcinoma [6]. Response to taxane/platinum in clear cell carcinoma is still controversial. Reed et al. [7] suggests that common resistance mechanism might be a central determinant for response to current combination therapy regardless of histologic type.

The cytoprotective chaperone protein, clusterin (CLU), has been reported to be involved in numerous physiological processes important for carcinogenesis and tumor growth, including apoptotic cell death, cell cycle regulation, DNA repair, cell adhesion, tissue remodeling, lipid transportation, membrane recycling, and immune system regulation [8]. CLU protein is commonly up-regulated by chemotherapy and radiotherapy in cancer cells, and contributes to cancer cell resistance *in vitro *and in various animal models of cancer by blocking apoptosis [9]. Cytoplasmic CLU is consistently reported to be associated with chemoresistance and it is present in a wide range of advanced cancers as shown in human tumor tissues from prostate [10,11], renal [12], breast [13], ovarian [14], colon [15], lung [16], pancreas [17], cervix [18], melanoma [19], glioma [20], and anaplastic large cell lymphoma [21].

Recent clinical trials using OGX-011, an antisense oligodeoxynucleotide specifically targeting CLU by complementing CLU mRNA translation initiation site have been launched [22]. OGX-011 potently inhibits CLU expression and enhances the efficacy of anticancer therapies in vitro and in vivo [23,16]. In addition to a phosphorothioate backbone, OGX-011 incorporates a 2'-methoxyethyl modification to the ribose moiety on the flanking four nucleotides. This allows formation of RNA duplexes with higher affinity for improved potency, increases nuclease resistance prolonging tissue half-life [24] and decreases toxicity with less nonspecific immune stimulation than unmodified phosphorothioate antisense [25].

In ovarian cancer, very limited number of studies has directly examined the effect of altering CLU expression on cell death and survival. Thus, prognostic significance of CLU expression in ovarian cancer patients remains controversial [26-29]. To establish the clinical significance of CLU as a potential molecular target to predict survival in ovarian cancer patients, we conducted this study.

## Methods

### Cell line

Human ovarian cancer cell line, KF, was provided as a generous gift by Dr. Yoshihiro Kikuchi, National Defence Medical College, Saitama, Japan. Another ovarian adenocarcinoma cell lines, SKOV-3 and OVK-18 cells, were purchased from ATCC, and clear cell carcinoma cell lines, KOC-7c and TU-OC-1, were provided as a generous gift by Dr. Junzo Kigawa, Tottori University, Japan. All cell lines were maintained in RPMI-1640 supplemented with 2 mM L-glutamine, and 10% FCS (Sigma, St. Louis, MO, USA) OVK18 cells, maintained in DMEM supplemented with 2 mM L-glutamine and 10% FCS (Sigma). Both KF-TX and SKOV-3-TX clone were established from parental cell lines KF and SKOV-3, respectively by maintaining each clone in increasing sublethal concentration of TX (up to 10 nM for KF-TX and 2 nM for SKOV-3-TX) for more than ten months then IC50 of each clone was determined by the viability assay after three days treatment.

### Antibodies and reagents

Mouse anti-human CLU (clone 41 D, Upstate Biotechnology, Lake Placid, NY, USA) was used at 1:1,000 dilution for western blotting. Immunoblotting detection was done with anti-mouse secondary horseradish-peroxidase-conjugated antibodies (Dako) diluted 1:2,000. TX was supplied by Bristol-Myers Co. Ltd. (Japan). We then prepared stock solution by diluting TX in the media at a final concentration of 4 μM and further working dilutions were carried out to reach the desired concentration. Antisense oligodeoxynucleotide against CLU (OGX-011) was provided by Oncogenex (Canada).

### Transient transfection of KF-TX cells with si-RNA or OGX-011

To knock down the expression of CLU, siRNA or OGX-011 was used in this study. Validated siRNA oligomers directed against the s-CLU mRNA leader endoplasmic reticulum signal peptide (s-CLU-siRNA) [30] and a control sequence which does not match any gene sequence (Cont-siRNA) were synthesized by Ambion (USA): s-CLU-siRNA, 5-GCG UGC AAA GAC UCC AGAAdTdT-3 and 3-dTdTCGC ACG UUU CUG AGG UCU U-5; Cont-siRNA, 5-GCG CGC UUU GUA GGA UUC GdTdT-3 and 3-dTdTCGCGCG AAA CAU CCU AAG C-5. s-CLU-siRNA or cont-siRNA were transfected into ovarian cancer cells (10^5 ^cells/60-mm dish) using SiPORT *Neofex *(Ambion; USA) at a final concentration of 200 nM. KF-TX cells were cultured to 50% confluence. Transfection of OGX-011 was done twice using Effectine (Qiagen, Hilden, Germany) according to the manufacturer's instructions. Twenty four hours after last transfection, cells were treated with various concentration of TX for 72 h. Then cellular viability was evaluated.

### Plasmids

pIRES/hygro and pIRES/hygro-full CLU expressing vectors have been previously described [31]. Vector expressing short hairpin RNA against CLU RNA (CLU-shRNA; ver.3) was purchased from Upstate Biotechnology (Lake Placid, NY, USA).

### Generation of cell lines stably expressing s-CLU

OVK-18 cells were cultured to 50% confluence. Plasmid DNA transfection was done using Effectine (Qiagen) according to the manufacturer's instructions. pIRES-hygro or pIRES-CLU-hygro-transfected OVK18 cells were selected in hygromycin (50 μg/ml; Sigma). Selected colonies were screened by immunoblotting to identify stable clones expressing s-CLU.

### Cell viability assay

Cell viability was evaluated using cell counting kit (CCK-8) (Dojindo, Kumamoto, Japan). Briefly, transfected cells were pre-cultured in 96-well plate (3,000 cells/well) for 24 h. Seventy two hours after TX treatment at the indicated doses, culture media were replaced by the WST-8 reagent. Reduced WST-8 by the cellular dehydrogenases turns into orange formazan. Produced formazan is directly proportional to living cells. Absorbance was measured at 450 nm by microplate reader equipped by computer (NEC, Tokyo, Japan).

### Flow cytometry analysis

Following TX treatment, cells were trypsinized, washed twice in phosphate-buffered saline (PBS) and cell cycle phases were analyzed. Briefly, cells were fixed at 4°C overnight in 70% ethanol. After washing with Ca2+-Mg2+-free Dulbecco's PBS, cells were treated with 0.1 μg/ml RNase (Type I-A, Sigma), stained with 100 μg/ml propidium iodide (PI; Sigma) for 20 min, filtered and kept on ice until measurement. Cells were acquired by the FACS calibrator (BD, Bioscience) and then analyzed using the ModFit software (Verity software; ME, USA). Cell fractions with a DNA content lower than Go/G1, the sub-G0/G1 peak, were quantified and considered a marker of the number of apoptotic cells.

### Annexin V staining

After harvesting and washing as described above, the cells were stained directly with PI at final concentration of 10 μg/ml and 2% Annexin-V Flous (Roche, Basel, Swizerland) in incubation buffer (10 mM Hepes/NaOH, pH 7.4, 140 mM NaCl, 5 mM CaCl2) for 10 minutes. Cells were acquired with the FACS calibrator (BD) after setting the instrument with controls (non-treated, stained cells) after two washes in PBS. In this experiment, both cells with early apoptotic signals, stained with annexin V, and cells with late death signals, stained with PI, are all considered and quantified. Apoptotic cells were analyzed using the CellQuest software.

### Western blotting

Cell lysates were obtained by resuspending cells in RIPA buffer (10 mM Tris (pH 7.4), 150 mM NaCl, 1% Triton X-100, 1% Nadeoxycholate (Kanto Chemical, Tokyo, Japan) and 5 mM EDTA) supplemented with protease inhibitors cocktail (Sigma, USA). Protein concentration of subcellular fractions or whole cell lysates was determined by BSA assay using the BSA kit (Pierce, Rockford, IL), then equal protein amounts were heated to 100°C for 5 min with SDS sample buffer (25 ml glycerol, 31.2 ml Tris buffer, 7.5 ml SDS, a dash of bromophenol blue/100 ml) and run on 10% SDS-PAGE. Protein samples were then blotted onto PVDF membranes (Immobolin P, Watford, UK). The membranes were incubated in blocking solution (5% non-fat milk in PBS) for 1 h, then in primary antibody (anti-human CLU mAb at dillutin of 1:1000) overnight. After 3 × 10 min washes in TBS (0.1% Tween-20 in PBS) the membrane was incubated for 1 h at room temperature with horseradish peroxidase (HRP)-linked IgG (1:2,000 dilution in T-TBS) followed by three washes (10 min each) with T-TBS. Signal on membranes was developed using ECL reagent (Amersham, USA) and then was imaged with Polaroid imaging system (Amersham,USA).

### Immunohistochemistry

Immunohistochemical staining of CLU was performed as previously described [19,32]. Detection of CLU was performed using a commercial polyclonal anti-CLU antibody (alpha/beta rabbit polyclonal antibody H330: Santa Cruz Biotechnology, Santa Cruz, CA, USA). The CLU antibody was used at 1:200 dilution for overnight at 4°C. Negative control were obtained by omitting the primary antibody. All slides were blindly evaluated for CLU immunoreactivity and protein localization, without knowledge of clinicopathological data.

Immunohistochemistry was performed in eight pairs of primary and their recurrent matched tumors of ovarian cancer specimens. All samples used were obtained from surgically staged ovarian cancer patients. Primary surgery was performed with the intention of maximal debulking. The indication for secondary surgery was for single recurrent tumor or interval debulking or secondary debulking. All patients were treated with standard TC regimen intravenously (TX; 175 mg/m^2^, carboplatin; AUC5) as first line chemotherapy. In this study, chemo-responsive tumors were defined as tumors initially responding to front-line chemotherapy with no relapse for at least one year. Tumors showing no response or recurring within one year after the first treatment were defined as chemo-resistant.

For survival analysis, we divided 47 patients with early-stage ovarian cancer into two groups based on scoring as previously described [19]. All patients received complete surgical staging and TX/platinum-based adjuvant chemotherapy except stage Ia, non-clear cell carcinoma.

### Statistical evaluation

For in vitro experiments, statistical analyses were performed using Minitab Release (Ver.12). Data are expressed as mean ± S.E.M. One-way analysis of variance was used to assess statistical significance between means. Differences between means were considered significant if p-values less than 0.05.

For statistical analysis of immunohistochemical expression of CLU, correlation between the variables and CLU immunoreactivity was analyzed using chi square test or Fisher's exact test. Patients' survival was calculated using Kaplan-Meier method. The significance of the survival difference was examined by the log-rank test. P < 0.05 was considered statistically significant. Statistical analyses were performed with the Statview software package (SAS Institute, Inc, Cary, NC).

## Results

### CLU was upregulated in chemoresistant ovarian cancer tissues

**Figure 1:**
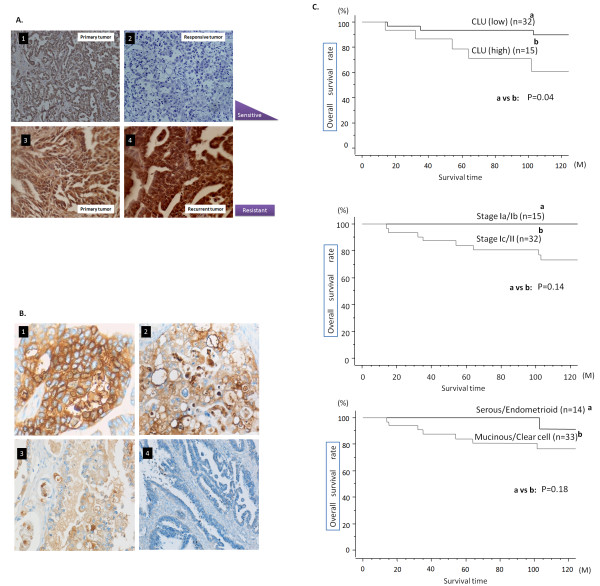
**Immunohistochemical detetion of CLU in ovarian cancer tissue samples A**. Representative images from immunohistochemistry detection of CLU expression in primary tumor specimens from chemo-responsive tumor tissues (1). CLU staining is moderate or very low. Recurrent tumor from the same patient also showed extremely limited staining of CLU (2). CLU staining in the primary tumor from chemo-resistant tumor tissue (3) showed high CLU expression. Recurrent tumors from the same patients, however, showed high CLU expression after chemotherapy (4).B. Representative photos of immunohistochemical expression of CLU in 47 tissue samples of ovarian cancer. 1) high expression, 2) moderate expression, 3) low expression, and 4) negative expression. C. Kaplan-Meier survival curve according to CLU expression (1), stage (2) and histology (3). Survival of patients with high and moderate expression of CLU showed significantly poor survival than that of low and negative expression of CLU (p = 0.04).

In a pilot experiment to check the relationship between CLU overexpression and chemoresistance in clinical samples from ovarian cancer patients, we performed immunohistochemistry using CLU Ab. Table 1 summarized CLU expression in eight primary ovarian cancer specimens together with their recurrent matched tumors. Importantly, primary chemo-responsive tumors showed either very limited or moderate CLU expression while CLU expression decreased in the recurrent tumors from same patients after chemotherapy course (Figure 1A.1,.2, respectively). In contrast, primary tumor samples from chemo-resistant cancers showed either high or moderate CLU expression in the primary tumor, and CLU expression was still high or up-regulated in the recurrent tumors (Figure 1A.3,.4, respectively).

**Table 1:** Clusterin expression pattern in the primary and recurrent ovarian cancers

Case (patient's age)	Chemo-senitivity	primary tumor	Persistent/recurrent t.	histology	FIGO stage
				
		CLU intensity	CLU intensity		
1 (57)	responsive	++	+	serous	IIIc
2 (48)	responsive	++	+	serous	IIIc

3 (48)	resistant	+	++	serous	IV
4 (53)	resistant	+	+++	serous	IV
5 (59)	resistant	+	+++	serous	IV
6 (52)	resistant	++	+++	serous	IIIc
7 (51)	resistant	N	++	serous	IV
8 (55)	resistant	+++	+++	serous	IIIC

N denotes negative staining, (+) denote weak staining (++) denote moderate staining, while (+++) denotes strong staining.

### Correlation of CLU expression with clinicopathological factors and survival in the patients with early-stage ovarian cancer

To assess any relationship between CLU expression and clincopathological characteristics of ovarian cancer, we used ovarian cancer tissue specimens from 47 patients with early-stage ovarian cancer. Fifteen patients had stage Ia disease, twenty one stage Ic, one stage IIa, one stage IIb and nine stage IIc. Clinicopathological characteristics of the patients were shown in Table 2. Immunolocalization with anti-CLU antibody largely showed positive staining in the cytoplasm of cancer cells and occasionally positive in the nucleus (Figure 1B). Among early stage ovarian cancer patients who underwent complete cytoreductive surgery including systematic pelvic and para-aortic lymphadenectomy, the association between the expression of CLU protein in ovarian cancer tissues and several clinicopathological factors revealed that age (p = 0.83), histologic subtype (p = 0.32) were not related to CLU expression, while FIGO stage showed the relation to CLU expression with marginal significance (p = 0.09) (Table 3). The estimated 5-year survival rate was 93.6% for patients with low CLU expression (n = 32), 78.8% for those with high CLU expression among early stage patients (n = 15; Figure 1C.1). There was a statistically significant difference of survival between the groups (p = 0.04). Age (p = 0.65), FIGO stage (5-year survival rate was 100% for stage Ia/Ib (n = 15) and 84.0% for stage Ic/II (n = 32); p = 0.18), and histological subtype (survival rate was 100% for serous/endometrioid (n = 14); and 84.2% for mucinous/clear cell (n = 33); p = 0.14), which were not related to poor survival in this patient cohort. (Figure 1C2, 1C3 and Table 3).

**Table 2:** Clinicoparhological characteristics of patients with early-stage ovarian cancer

Factor	n	%
**Age**		
<50	24	51.1
> = 50	23	48.9
**Histology**		
Serous/endometrioid	14	29.8
Mucinous/clear cell	33	70.2
**Stage**		
Ia/b	15	31.9
Ic/II	32	68.1

**Table 3:** Association between CLU expression and clinicopathological factors in early-stage ovarian cancer

Factor	CLU expression		P-value
		
	Low	high	
**Age**			0.83
<50	16	8	
> = 50	16	7	
**Histology**			0.32
Serous/endometrioid	11	3	
Mucinous/clear cell	21	12	
**Stage**			0.09
Ia/b	13	2	
Ic/II	19	13	

### CLU is upregulated in chemoresistant ovarian cancer cell lines

**Figure 2:**
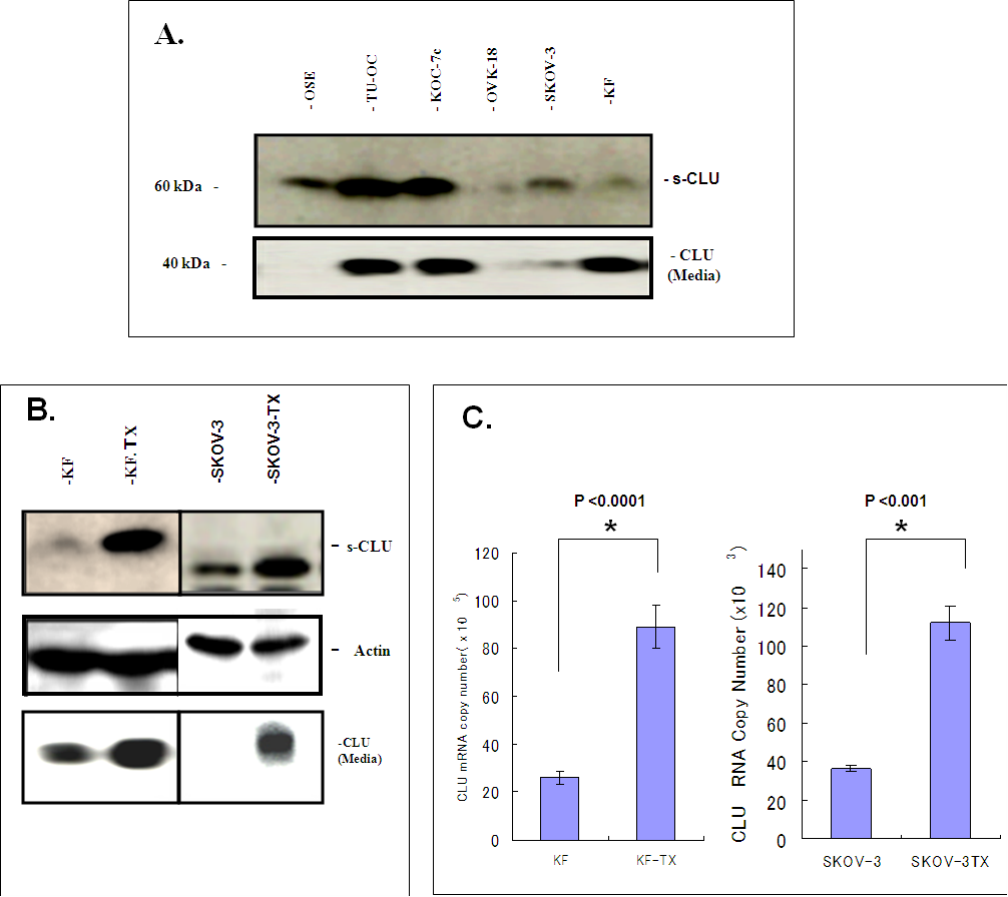
**S-CLU is up-regulated in the chemo-resistant cells**: A. Western blotting analysis of CLU in a panel of ovarian cancer cells. Equal amount of proteins were loaded, resolved by SDS-PAGE and immunoprobed with anti-CLU mAb. S-CLU was found in the cells and media. Some ovarian cancer cells express relatively high levels of CLU in comparison to immortalized non tumorigenic ovarian epithelial OSE cells. B. Chemo-resistant KF-TX cells shows higher expression levels of CLU compared to parental KF cells. A similar result is found in SKOV-3 compared to SKOV-3-TX cells. C. Quantitative PCR showing the difference in CLU transcript level between the TX-sensitive and TX-resistant clones in both KF (left) and Skov-3 (right) systems.

To verify our observation in the ovarian cancer cell lines we further analyzed CLU expression in a panel of ovarian cancer cell lines with different response pattern to TX (different IC50) by western blot, revealed that all the ovarian cancer cell lines showed moderate or high CLU expression with the exception of OVK-18 cells, which showed limited CLU expression. S-CLU expression was relatively higher in cell lines with high IC50 of TX (Figure 2A and Table 4). We then established KF-TX cells (IC50 = 500 nM) from parental KF cells (IC50 = 100 nM; see materials and methods). Importantly, KF-TX showed higher expression of s-CLU in comparison with parental KF cells (Figure 2B). To verify whether increased s-CLU expression correlates with TX resistance was not unique to KF cells, we similarly established SKOV-3-TX (TX-resistant) from responsive parental SKOV-3. Over-expression of s-CLU was also observed in SKOV-3-TX compared to SKOV-3 (Figure 2B). Quantitative RT-PCR confirmed enhanced expression of s-CLU strictly correlated to mRNA expression in both KF-TX and SKOV-3-TX cells when compared with their parental cell lines (Figure 2C).

**Table 4:** List of ovarian cancer cells and their IC50 for TX in a three days treatment experiment.

Histological type	Cell line	IC50 (TX; nM)
Serous	KF	100
Serous	KF-TX	500
Serous	SKOV-3	20
Serous	SKOV-3-TX	100
Serous	OVK18	50
Clear cell	TU-OC-1	6900
Clear cell	KOC-7c	6700

**Figure 3:**
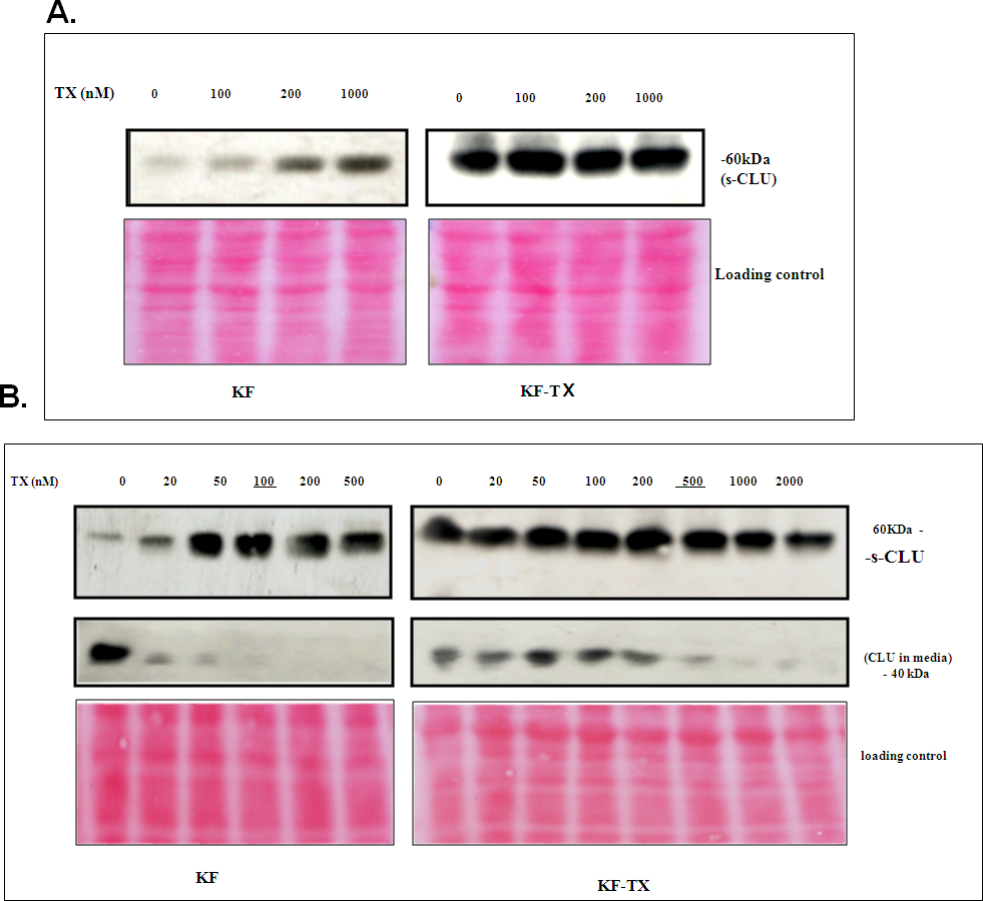
**Induction of CLU in a time and dose dependent fashion by TX**. A. Western analysis showing s-CLU expression after 6 h treatment with TX. Induction of s-CLU is evident in chemo-sensitive KF cells when treated with high doses of TX but not in KF-TX, in which the high levels of s-CLU remained unchanged. Under these conditions, KF cells' viability decreased. B. Western analysis showing s-CLU expression in cell extracts (upper panel) and culture media (lower panel) after 48 h treatment with TX. CLU increased in TX-sensitive KF cells at different doses while CLU secretion was inhibited. At difference, expression and secretion of CLU was unchanged in the TX-resistant cells. Only at very high concentrations of TX a consistent down-regulation of s-CLU in the media was detectable. Ponceau S staining of the blot is provided to show equal loading of the protein samples because Actin and tubulin are responding to TX. The data shown are representative of four independent experiments.

To investigate whether upregulation of s-CLU expression is a cause or a result of TX-induced resistance, both parental KF and KF-TX cells were treated with TX in a dose dependent fashion for 6 h. Sensitive KF cells rapidly responded by increasing s-CLU expression level under low doses of TX. In this experiment cellular viability mainly decreased when TX dose surpassed IC50. KF-TX cells already expressing higher CLU levels, did not further express CLU following TX treatment (Figure 3A). When we treated cells with TX up to 48 h, KF parental cells progressively increased CLU expression levels up to IC50 doses (100 nM) then CLU was down-regulated at higher doses. On the other hand, CLU expression level (already high) did not change in KF-TX cells. Again, only at doses higher than IC50 (500 nM), CLU was down-regulated (Figure 3B). S-CLU detected in cells' medium progressively decreased up to IC50 doses in the sensitive cells suggesting its retention inside cells. However, secretion of CLU into the media by resistant cells clearly extended up to higher concentration of TX if compared with parental cells. Considering that changes in CLU expression seem independent of CLU mRNA, which did not change significantly as indicated by real-time PCR (data not shown), these results suggest that post-translational modification of CLU, including maturation and secretion, may be altered in response to TX treatment.

### Overexpression of s-CLU confers resistance to TX in vitro

**Figure 4:**
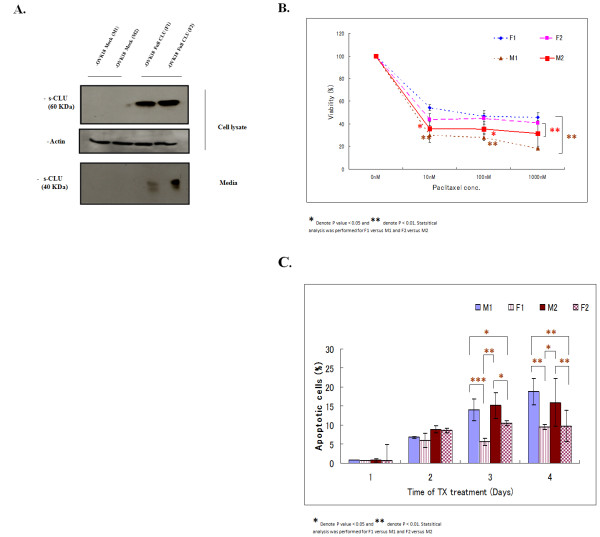
**Over-expression of CLU confers TX-resistance to OVK18 cells**. A.Western blotting analysis showing the expression level of s-CLU and mature secreted (40 kDa) CLU in the media in two recombinant OVK18 survivor clones F-1 and F-2 compared with two mock clones M-1 and M-2. The pIRES-hyg-full-length-CLU cDNA expression vector was used for transfection experiments (see Materials and Methods). S-CLU was only detectable in the media of F-1 and F-2 clones. B. Comparison of relative viability of clones F1 and F2 with regard to mock clones M1 and M2 in the presence of different doses of TX. F-1 and F-2 clones show significantly increased viability. Each data point represents the mean of three experiments; bars denote SD; * indicates difference from mock at P < 0.001. C. Quantification of the relative proportions of apoptotic cells by FACS analysis of M-1 and -2 and F-1 and -2 clones in a time-course experiment. Cells were counted, divided into groups in triplicates and challenged by TX at 100 nm for the indicated time periods. Cells were then acquired by FACS calibrator and the apoptotic sub-diploid peak was analyzed and quantified using the Cell-quest software. Significant inhibition of TX-induced apoptosis was observed in the clones stably expressing CLU (F-1 and F-2).

To confirm the cytoprotective role of s-CLU *in vitro*, we established two cell clones stably expressing full-length CLU (a gene able to express s-CLU) from the OVK18 cells with low endogenous CLU, OVK18-s-CLU-1 (F-1) and OVK18-s-CLU-2 (F-2). As shown in Figure 4A, very limited endogenous CLU is expressed and secreted by parental OVK18 cells, while CLU is detectable in both F-1 and F-2 clones as precursor and secreted form in cell extract and media. When cell viability of both clones was assayed under progressively increasing TX doses, it was significantly higher than mock controls (M-1 and M-2 (p < 0.05; Figure 4B)). Figure 4C summarizes the result of FACS analysis of F-1/F-2 clones compared to M-1/M-2. F-1 and F-2 showed a significantly lower cell death as assessed as sub-diploid peak, under TX stress when compared to M-1 and M-2. These data confirmed the cytoprotective effect of s-CLU in ovarian cancer cells.

### SiRNA against s-CLU and OGX-011 modulates sensitivity to TX in KF-TX cells

**Figure 5:**
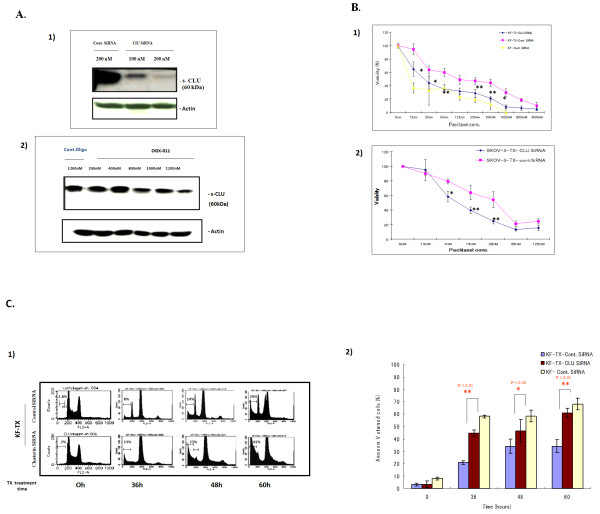
**Targeting CLU by siRNA or OGX-011 sensitizes ovarian cancer cells to TX treatment**. A. Western blotting showing the efficacy of siRNA transfection or OGX-011 in s-CLU depletion in KF-TX cells. (1) CLU expression after two sequential transfections with siRNA against CLU (see materials and methods) at 100 and 200 nM are compared with control siRNA at 200 nM. Transfection at 200 nM knocked down about 90% of target CLU (far right panel). The basic expression level without any transfection had not been affected neither by transfection reagents (data not shown) nor by control siRNA transfection (far left panel). (2) CLU expression after two sequential transfections with OGX-011 (see materials and methods) at 200-1200 nM are compared with control oligonucleotides. OGX-011 transfection at 800, 1000 and 1200 nM significantly knocked down CLU expression (far right panels). B. Comparative viability of different ovarian cancer cells before and after CLU knock down are. Cells were cultured in 96-well plates, then transfected either with CLU-siRNA or control siRNA twice. Twenty-four hours after last transfection, cells were treated with TX. Seventy-two hours after drug addition at indicated doses, cell viability was estimated. Both KF-TX cells (1) and SKOV-3-TX (2) showed enhanced TX-induced toxicity in CLU KD cells versus controls. C. Time-dependent FACS analysis demonstrating that CLU-siRNA enhanced TX toxicity in KF-TX cells. KF-TX cells were transfected either with CLU-siRNA or control siRNA and challenged with TX dose of 200 nM at indicated time periods. Representative DNA histograms show the apoptotic response to TX with and without CLU-siRNA transfection (1). Annexin V staining of cells treated as in panel (1). Time-course quantification of the relative ratio of apoptotic cells at different time points in the presence of CLU siRNA or controls when cells were challenged with TX (2).

To determine whether s-CLU protects ovarian cancer cells from TX-induced cell death or not, siRNA oligomers specific for exon II of CLU mRNA and OGX-011 were used to knock down CLU expression. TX resistant ovarian cancer cells, KF-TX, were transfected either with siRNA or OGX-011. CLU gene mRNA was amenable to siRNA transfection at doses of 100 and 200 nM (Figure 5A.1) and to OGX-011 at doses of 400, 800, 1000 and 1200 nM as well (Figure 5A.2). We then considered 200 nM of siRNA and control siRNA and 1200 nM of OGX-011 and control oligodeoxynucleotide to be used in further experiments because they maximally reduced CLU expression.

To evaluate the benefits of targeting s-CLU in sensitizing ovarian cancer cells to TX, cellular viability of KF-TX under a dose dependent fashion of TX treatment was studied in both CLU-siRNA and control-siRNA (cont-siRNA) transfected cells. Under these experimental conditions Figure 5B.1 shows significant reduction in cell viability of KF-TX, pre-treated with CLU-siRNA, under different doses of TX than those pre-treated with control-siRNA then TX. Similarly, CLU knocking down by siRNA significantly sensitized SKOV-3-TX cells (Figure 5B.2).

Moreover, sensitization to TX in KF-TX cells by CLU-siRNA was further confirmed after time dependent fashion of TX treatment by FACS analysis (at 36, 48 and 60 h; Figure 5C.1) where dead cells indicated by the sub diploid G0 cells. Further confirmation for differential apoptotic cells was obtained by Annexin V staining (Figure 5C.2). These data indicate that response to TX administration is enhanced after CLU-siRNA transfection. In addition, a dose dependent enhancement of apoptosis by TX in KF-TX cells after CLU knock-down was verified by DNA laddering experiment (data not shown).

**Figure 6:**
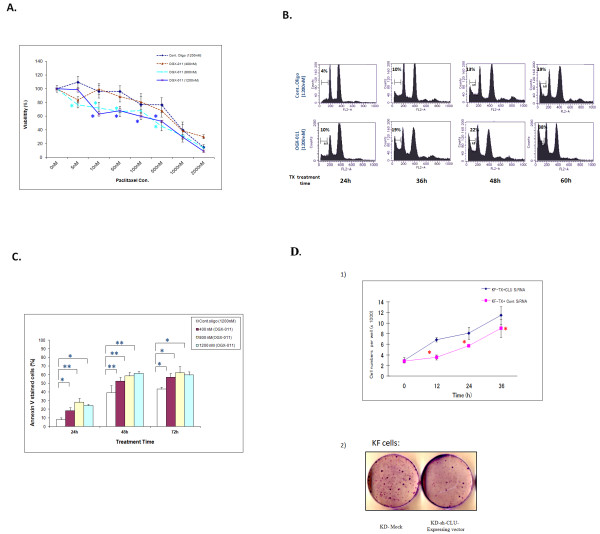
**Targeting CLU by OGX-011 sensitizes ovarian cancer cells to TX treatment**. A.Comparative viability of chemoresistant ovarian cancer cells before and after CLU knock down by OGX-011. Cells were cultured in 96-well plates, then transfected either with CLU-siRNA or control siRNA twice. Twenty-four hours after last transfection, cells were treated with TX. Seventy-two hours after drug addition at indicated doses, cell viability was estimated. KF-TX cells showed enhanced TX-induced toxicity in CLU KD cells versus controls. B. A representative time-dependent DNA histogram (FACS analysis) demonstrating that CLU KD by OGX-011 at 1200 nM enhanced TX toxicity in KF-TX cells. KF-TX cells were transfected either with OGX-011 or control Oligonucleotide and then challenged with TX dose of 200 nM at indicated time periods (24 h, 36 h,48 h and 60 h). B. Results of Annexin V staining of cells pre-treated with OGX-011 at different concecntrations (400, 800 and 1200 nM) then treated with TX (200 nM) for indicated time periods (24, 48 and 72 h). Quantification of the relative ratio of apoptotic cells at different time points indicated the significant enhancement of TX toxicity by OGX-011. The maximum enhancement was obtained by 800 nM OGX-011 while the conc. of 1200 nM did not show further significant improvement in toxicity. D. CLU knock down modulates cellular growth rate of ovarian cancer cells. (1) KF-TX cells showed enhanced growth rate when transfected with CLU siRNA with regard to controls. KF-TX cells either pre-transfected twice with CLU siRNA or control siRNA and then divided in 24 well plat in triplicates. Cell number was counted manually each 12 h (2). Representative clonogenic assay shows that targeting CLU by siRNA (sh-CLU) increased TX-induced clonogenic toxicity in KF cells. In this case, KF cells were either transfected with CLU short hairpin expressing vector (CLU-shRNA) or mock control alone and then cells were challenged by increasing doses of TX starting from 2-5 nM for three weeks. The resistant colonies surviving drug stress were stained by Giemsa after methanol fixation and pictures were taken with a digital camera..

On the other hand, cellular viability was studied under experimental conditions similar to this described above except that OGX-011 was used to knock down CLU while control oligodeoxynucleotide was used for control transfection. Figure 6A shows significantly less viability of KF-TX cells pre-treated with OGX-011 and TX than those pre-treated with control oligodeoxynucleotide and TX. Similarly, sensitization to TX in KF-TX cells by OGX-011 was further confirmed by FACS analysis (Figure 6B). Further confirmation for differential apoptotic cells was obtained by Annexin V staining (Figure 6C). Together, the aforementioned data indicate that silencing s-CLU by specific siRNA or OGX-011 enhanced TX toxicity in the ovarian cancer cells.

### Knock-down of s-CLU enhanced cellular growth rate in KF-TX and reduced clonogenic ability in parental KF cells

To understand more about how s-CLU contribute to the fate of ovarian cancer cells, cellular growth rate following CLU-siRNA transfection was studied in KF-TX cells. Under these conditions, growth rate of KF-TX cells with CLU knock-down significantly increased compared with control siRNA-transfected cells (Figure 6D.1). Moreover, we established stable CLU-silenced cell system using CLU short hairpin expression vector (CLU-shRNA) in KF parental cells to study the effect of stable knock down of CLU on the long treatment of TX. Under these conditions, we proceeded to TX treatment with sub-lethal but increasing doses (2-10 nM of TX) for three weeks. Then, clonogenic ability over TX administration was studied. Importantly, CLU-shRNA significantly reduced the generation of TX-resistant clones if compared with mock transfectants (Figure 6D.2) indicating that s-CLU expression is necessary for ovarian cancer cells to develop TX resistance probably to inhibit cell growth.

## Discussion

In the present study, we have shown that CLU expression is a prognosticator for ovarian cancer patients who were treated with primary complete surgical staging and adjuvant taxane/platinum combination chemotherapy in early-stage disease. Prognostic significance of CLU expression has been reported in different cancer types in the literature. The expression level of CLU in renal cancer cells was found to be closely associated with pathological stage and grade of the tumor; and the overall and recurrence-free survival rate of patients with strong CLU expression was significantly lower than that of patients with weak expression [33]. CLU expression levels correlated with tumor size, estrogen and progesterone receptor expression levels, and lymph node metastasis in breast carcinoma [32]. Similarly, CLU has been proposed to be a new potential prognostic and predictive marker for colon carcinoma aggressiveness, since overexpression of CLU is observed in highly aggressive tumors as well as metastatic nodules [15]. However, prognostic significance of CLU expression remains controversial for ovarian cancer patients. Recent publication described that the average survival time of the patients with CLU overexpression was significantly shorter than those with normal CLU expression [26]. CLU expression was associated with survival of patients with primary ovarian cancer (relative risk for overall survival 1.69; 95% confidence interval, 1.52 to 1.95 (P = 0.033)). On the contrary, it was reported that high expression of CLU was related to favorable prognosis in advanced-stage (stage III) serous ovarian cancer [28]. Although our observations were consistent with previously reported ones that s-CLU mediates cisplatin-induced resistance in ovarian cancer [34], CLU expression was found not to be a prognostic factor among patients with advanced-stage (stage III/IV) ovarian cancer in our patient cohort (data not shown). Moreover, our study showed that s-CLU is well expressed in many ovarian cancer cell lines assayed and resistant ovarian cancer tissues. Additionally, through mechanisms not yet elucidated, as a consequence of acquired resistance, CLU biosynthesis is altered and up-regulated in ovarian cancer cells.

Optimal surgery is a strong prognosticator for advanced-stage ovarian cancer, which was also found in our advanced-stage patient cohort (data not shown), and it is widely accepted that complete cytoreduction is the most important prognostic factor for ovarian cancer. We found that immunohistochemical expression of CLU showed prognostic significance for the patients with early-stage (stage I/II) patients who underwent complete cytoreduction as a primary surgery, whereas histologic subtype and stage are not associated with their survival. Perhaps, the response to front-line chemotherapy might be one of the most important factors for survival among the patients with early-stage disease. Our result suggests that CLU is related to survival because overexpression of CLU is related to chemoresistance [35,36]. That is might be because CLU can result in impaired survival for early-stage cases [26]. Alternatively, overexpression of CLU might increase migration and invasion capacity of ovarian cancer cells [27].

To improve the survival of ovarian cancer patients, we need to develop new combination therapy of cytotoxic drugs better than current standard regimen (TX/carboplatin; TC). However, the result of GOG182 to find superior regimen to TC was negative, indicating that it might be quite difficult to find new useful combination therapy better than TC [37]. Thus, it is necessary to test the efficacy of molecular targeting drugs such as bevacizumab with or without cytotoxic agents, or the new drugs to modulate sensitivity to platinums and/or taxanes for better survival.

S-CLU expression had changed upon acquisition of TX-resistance and TX treatment in ovarian cancer cells and tissues. SiRNA or OGX-011 administration caused efficient depletion of CLU mRNA in vitro. Under these conditions, TX stress induced apoptosis more efficiently in CLU-depleted cells most probably because of enhanced growth rate after s-CLU knock-down which makes cells rapidly trapped in the G2/M arrest by TX as a microtubule stabilizing agent. S-CLU may act as a cytoprotective protein [38] and also possesses extracellular chaperone-like activity inducing phagocytosis by nearby cells [39,9]. In this study, chemo-sensitivity induced by CLU gene silencing was directly correlated to the endogenous level of CLU protein expressed in a given cell line, being particularly enhanced in KF-TX, SKOV-3-TX, that express the highest levels of s-CLU. An experimental system in which OVK18 cells were genetically modified to specifically over-expression s-CLU rendered cells TX-resistant. Thus, in our system s-CLU seems essential for ovarian cancer cells to resist TX. Similar results have been obtained in cervical cancer [40]. Thus, up-regulation of s-CLU might be a candidate marker to predict ovarian cancer chemo-resistance, while its reduction after drug administration may predict chemo-response when tumor cells have high endogenous CLU.

Importantly, our results support the idea that, s-CLU is a stress-associated cytoprotective protein that is up-regulated in an adaptive cell survival manner following various cell death trigger including chemotherapy in ovarian cancer cells as well as in most cancer cells [41,35]. Therefore, novel therapeutic strategy of silencing s-CLU expression to overcome chemoresistance were suggested when cancer cells over-express s-CLU as in lung [42], prostate [43], kidney [44] or breast [13]. In the current study, we firstly demonstrated that OGX-011, a second-generation antisense oligodeoxynuclotide targeting the translation initiation site of human CLU gene exon II with a long tissue half-life, can modulate sensitivity to TX in an acquired TX-resistant ovarian cancer cell line. OGX-011 improved the efficacy of chemotherapy, radiation, and hormone withdrawal by inhibiting expression of CLU and enhancing apoptotic rates in preclinical xenograft models of prostate, lung, renal cell, breast, and other cancers [44-46].

Interference with the innate apoptotic activity is a hallmark of neoplastic transformation and tumor formation. Modulation of the apoptotic cascade has been proposed as a new approach for the treatment of cancer. Phenoxodiol [47] and XIAP inhibitor [48] are currently tested in clinical trials as chemosensitizer for chemoresistant tumors [49]. recently reported the result of the phase II study of docetaxel and prednisone with or without OGX-011 in patients with metastatic castration-resistant prostate cancer. They have shown that combination of OGX-011 with docetaxel significantly improved survival [49]. We do hope to test the efficacy of OGX-011 as a chemosensitizer to standard cytotoxic drugs for the patients with recurrent (resistant tumor) and refractory ovarian cancer.

## Conclusions

In summary, present study demonstrated that alterations of s-CLU biogenesis are induced during development of TX-resistance. These changes include overexpression inside cells and subsequent secretion into media positively correlates to chemo-resistant phenotype. Immunohistochemical detection of overexpression of CLU in early-stage (stage I/II) ovarian cancer tissues was significantly related to poor survival, while none of other clinicopathological factors analyzed were related to survival. We conclude that CLU could be a potential molecular marker to predict chemoresistance in patients with ovarian cancer. Thus, CLU gene seems to be a key element regulating chemo-response/chemo-resistance to TX. This gene product might be a potential therapeutic target to overcome the resistance to TX and improve the subsequent survival in ovarian cancer patients.

## Competing interests

The authors declare that they have no competing interests.

## Authors' contributions

MH, HW and ST designed the study. MH and LW performed cell-based experiments and the chemo-sensitivity assays studies, collected the data, carries out the statistical analysis and wrote the draft. HW, MH and TM carried out the immunohistochemistry and managed the database of clinical and pathological information and participated in writing the paper. ST and NS critically revised the manuscript and acquired the grant. NS supervised the experiment, acquired the grant and revised the final version. All authors read and approved the final manuscript.
